# Hypoxia-related tumor environment correlated with immune infiltration and therapeutic sensitivity in diffuse large B-cell lymphoma

**DOI:** 10.3389/fgene.2022.1037716

**Published:** 2022-10-14

**Authors:** Chen Liu, Lin Liu

**Affiliations:** Department of Hematology, First Affiliated Hospital of Chongqing Medical University, Chongqing, China

**Keywords:** hypoxia, diffuse large B-cell lymphoma, prognosis, immune cell infiltration, therapeutic response

## Abstract

**Background:** Due to the high heterogeneity of diffuse large B-cell lymphoma (DLBCL), traditional chemotherapy treatment ultimately failed in one-third of the patients. Big challenges existed in finding how to accurately predict prognosis and provide individualized treatment. Hypoxia, although being a key factor in the development and progression of DLBCL, plays its role in DLBCL prognosis, which has yet to be fully explored.

**Methods:** Data used in the current study were sourced from the Gene Expression Omnibus (GEO) database. DLBCL patients were divided according to different hypoxia-related subtypes based on the expressions of hypoxia-related genes (HRGs) relevant to survival. Differentially expressed genes (DEGs) between subtypes were identified using the limma package. Using univariate Cox regression and least absolute shrinkage and selection operator (LASSO) analyses, the prognostic signature was established to calculate risk scores. The tumor microenvironment (TME) in low- and high-risk groups was evaluated by single-sample gene set enrichment analysis (ssGSEA) and ESTIMATE. The chemotherapeutic sensitivity in two groups was assessed by IC50 values.

**Results:** DLBCL patients were clustered into two hypoxia-related subtype groups according to different gene survival and expressions associated with increasing oxygen delivery and reducing oxygen consumption, and these two subtype groups were compared. Based on the differential expression, a risk model was established using univariate cox and LASSO regression analyses, FNDC1, ANTXR1, RARRES2, S100A9, and MT1M. The performance of the risk signature in predicting the prognosis of DLBCL patients was validated in the internal and external datasets, as evidenced by receiver operating characteristic (ROC) curves. In addition, we observed significant differences in the tumor microenvironment and chemotherapeutic response between low- and high-risk groups.

**Conclusion:** Our study developed novel hypoxia-related subtypes in DLBCL and identified five prognostic signatures for DLBCL patients. These findings may enrich our understanding of the role of hypoxia in DLBCL and help improve the treatment of DLBCL patients.

## Introduction

Diffuse large B-cell lymphoma (DLBCL) is the most common type of non-Hodgkin lymphoma (NHL), which has great heterogeneity in clinical manifestations, histological morphology, and prognosis (De Paepe and De Wolf-Peeters, 2007; Sehn and Salles, 2021). It accounts for 31% of NHL cases in Europe and the United States and more than 40% of NHL cases in Asia (Barbui et al., 2018). Rituximab combined with cyclophosphamide, doxorubicin, vincristine, and prednisone (RCHOP) is the most common chemotherapy regimen, and there are still about 40% of patients who relapse eventually (Feugier et al., 2005; Pfreundschuh et al., 2006). With the deeper insight into fields of molecules, immunophenotypes, mechanisms, and tumor microenvironment, more and more chemotherapies and immunotherapies are emerging. Therefore, further elucidation of the molecular mechanism of DLBCL and development of new markers and therapeutic targets can provide new methods for treatment and intervention of DLBCL.

Hypoxia is a common feature in most tumors, including hematological malignancies. There is strong evidence that hypoxia influences the growth (Antebi et al., 2018; Tomecka et al., 2021), differentiation (Chen et al., 2018; Pattappa et al., 2019), and survival (Gomes et al., 2016) of many cell types which were cultured as monolayers and three-dimensional spheroids. Hypoxia-inducible factor-1α (HIF1-alpha) can promote the formation and recurrence of NHL by inducing angiogenesis *via* the VEGFA/VEGFRI axis (Minoia et al., 2013). The hypoxic microenvironment not only changes the metabolism of tumor cells but also the immune checkpoint of the tumor and enhances the ability of tumor immune escape (Koh et al., 2017a; Koh et al., 2017b). In addition, compared with normal tissues, tumor cells are more sensitive to reactive oxygen species (ROS) (Antony et al., 2017). Excessive ROS can also cause necrotic apoptosis of tumor cells. Screening of primary DLBCL patient samples revealed that the expression of enzyme hexokinase 2(HK2) was significantly correlated with the DLBCL phenotype, and genetic knockdown studies demonstrated that HK2 is required for promoting growth of DLBCL under hypoxic stress (Bhalla et al., 2018). Despite the importance of the hypoxic tumor microenvironment in B-cell development, little is known about the role of hypoxia in hematologic malignancies, including DLBCL.

In this study, we divided the data into two subtypes by consistent clustering at first. By identifying the differentially expressed genes between the two groups, the hypoxia genes which are significantly related to the disease were screened out and a risk model was established, and the correlation between the risk model and clinical factors was explored. At the same time, the mechanism of immune infiltration related to diagnostic genes in DLBCL was studied. Finally, the potential chemotherapeutic drugs are predicted, which provides a certain theoretical basis for the treatment and prognosis prediction of DLBCL.

## Materials and methods

### Data source

The clinical information and mRNA expression data on 199 DLBCL patients in the GSE11318 dataset and 412 in the GSE10846 dataset were retrieved from the GEO database. A total of 510 hypoxia-related genes (HRGs) were extracted in the UniProt database, as previously reported (Li et al., 2021a).

### Identification of hypoxia-related DLBCL subtypes

Using the GSE11318 dataset, 510 HRGs were input into univariate Cox regression analysis in order to identify survival-related HRGs with a *p*-value < 0.05. Then, those genes were included in consensus clustering analysis with parameter settings as maxK = 6, reps = 1,000, pItem = 0.8, clusterAlg = “km,” distance = “Euclidean,” and innerLinkage = “complete.” The t-SNE plot was carried out to evaluate the performance of consensus clustering. K–M curves were used to analyze the survival of patients in different hypoxia-related subtypes. In addition, the expressions of genes associated with increasing oxygen delivery and reducing oxygen consumption were extracted and compared between different subtypes.

### Identification of the prognostic signature in DLBCL

Differentially expressed genes (DEGs) between different subtypes were identified using the limma package with a *p*-value < 0.05 and |log_2_FC| > 1. The function of DEGs was analyzed by clusterProfiler, which comprised Gene Ontology (GO) terms, including those of the biological process (BP), cellular component (CC), and molecular function (MF), and Kyoto Encyclopedia of Genes and Genomes (KEGG) pathways. DLBCL patients from the GSE11318 dataset were then divided into training (n = 140) and internal testing (n = 59) sets. Univariate Cox regression was first applied to obtain DEGs significantly related to survival (*p*-value <0.05). Subsequently, the LASSO algorithm was applied to obtain the prognostic signature.

### Establishment of the risk score model and nomogram in DLBCL

Then, we determined the risk score using $\sum_{1}^{n}ExpGenei*Coefi$ . Based on the median of the risk score, DLBCL patients in the training set were divided into low- and high-risk groups. The survival in the low- and high-risk groups was evaluated by K–M curves. ROC curves were generated by the survival ROC package to assess the performance of the risk signature. Furthermore, the risk score model was tested in the GSE11318 internal testing set and GSE10846 external validation set. In addition, independent prognostic factors in DLBCL were screened by univariate and multivariate analyses. The nomogram was established to predict 1-, 3-, and 5-year survival of DLBCL patients. The calibration curves were plotted to assess the clinical use of the nomogram.

### Exploration of features in low- and high-risk groups

To characterize low- and high-risk groups, we 1) performed GSEA analysis to explore biological functions enriched in the two groups, 2) compared the tumor microenvironment *via* ESTIMATE and ssGSEA algorithms, 3) compared the chemotherapy (IC50) response using the pRRophetic package, and 4) compared the survival under the same stratification of clinical features.

### Statistical analysis

All data were analyzed by R software (version 4.0.0). The Kruskal–Wallis test was used for calculation in comparison among multiple groups. Comparisons among multiple groups were calculated by the Kruskal–Wallis test. A *p*-value < 0.05 was considered statistically significant unless otherwise specified.

## Results

### DLBCL patients were clustered into two hypoxia-related subtypes

**FIGURE 1:**
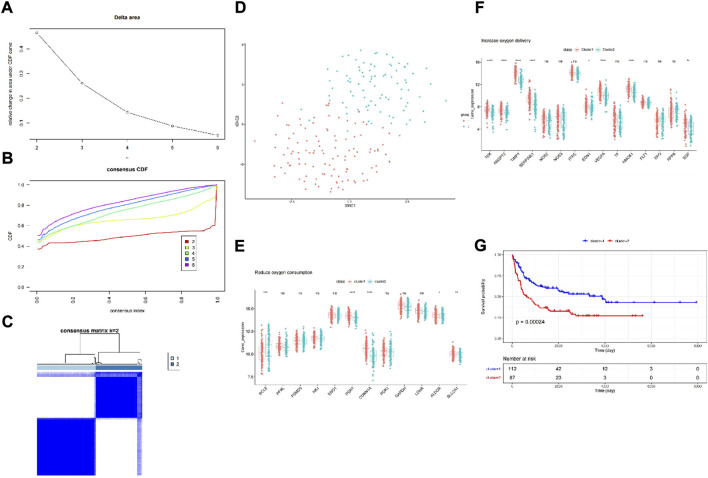
Consensus clustering identified two DLBCL clusters and differences in two hypoxia-related subtypes of DLBCL. **(A)** Relative change in area under CDF curve for k = 2–6; **(B)** Consensus clustering cumulative distribution function (CDF) for k = 2–6; **(C)** The DLBCL cohort from GSE11318 was divided into two distinct clusters when k = 2; **(D)** PCA of the GSE11318 dataset based on the expression profiles of the HRGs. **(E,F)** The expression of oxygen delivery-related genes and oxygen consumption-related genes between two hypoxia-related subtypes of DLBCL. **(G)** Kaplan-Meier survival analysis between two hypoxia-related subtypes of DLBCL. ns:not significant, * *p* < 0.05, ** *p* < 0.01, *** *p* < 0.001, **** *p* < 0.0001.

First, we chose GSE11318 as the training set and divided the expression of DLBCL patients into two groups (Figures 1A,D). According to the result of t-SNE and merging with the HRGs selected by univariate Cox regression analysis (Supplementary Table S1), we obtained increased oxygen delivery (Figure 1E) and reduced oxygen consumption (Figure 1F). Moreover, we observed that cluster 1 had better survival than cluster 2 (Figure 1G), indicating the importance of hypoxia in DLBCL.

### Five prognostic signatures were identified in DLBCL

**FIGURE 2:**
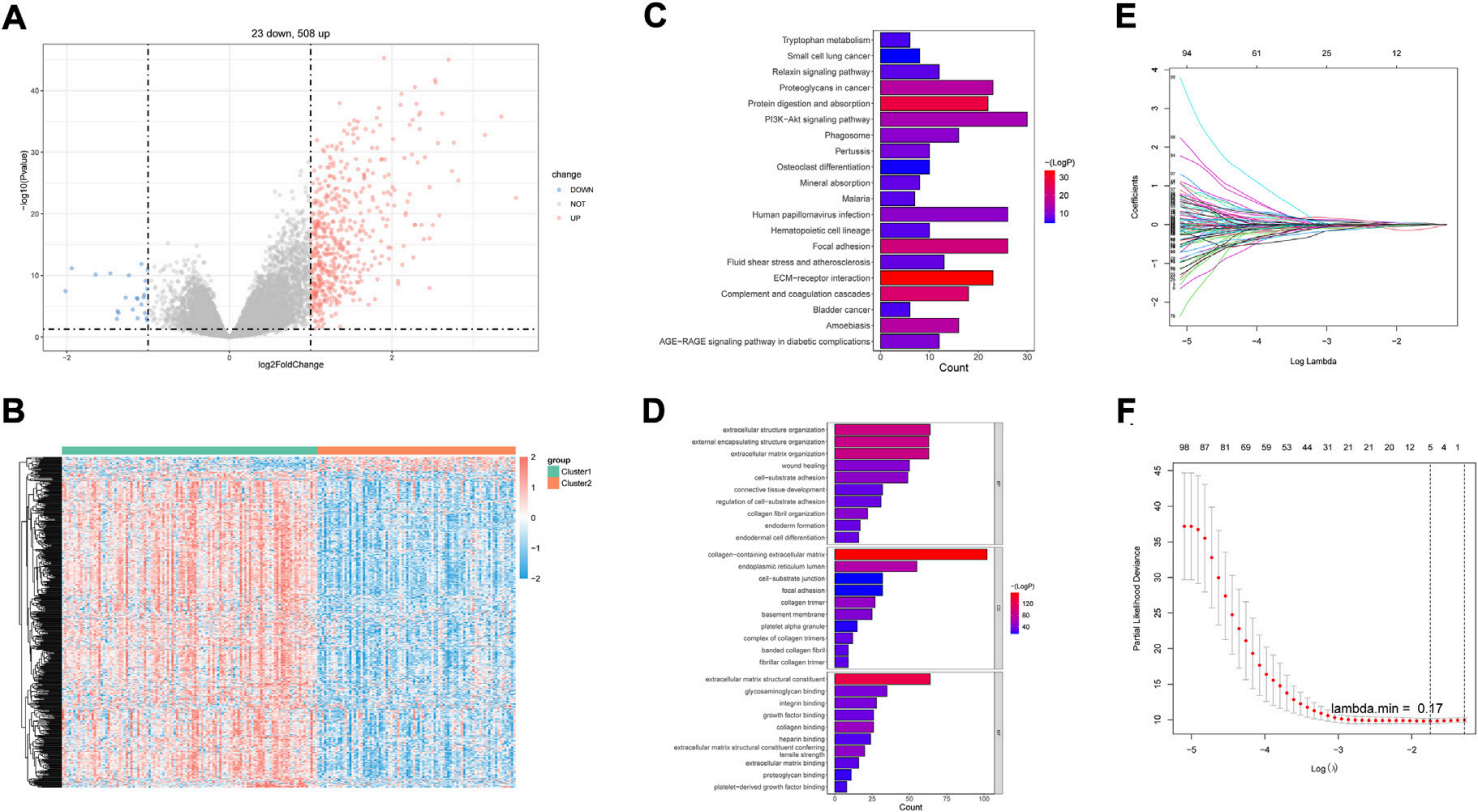
Identification of DEGs between two hypoxia-related subtypes of DLBCL and prognostic genes. P-value <0.05 was considered statistically significant. **(A)** Volcano plots for DEGs, **(B)** Heatmap of DEGs between two clusters. **(C)** Top20 KEGG pathways enriched by DEGs between two hypoxia-related subtypes of DLBCL. **(D)** Top10 GO terms enriched by DEGs between two hypoxia-related subtypes of DLBCL. **(E)** Least absolute shrinkage and selection operator (LASSO) coefficient profiles of five hypoxia-related DEGs. **(F)** Partial likelihood deviance for LASSO coefficient profiles.

**FIGURE 3:**
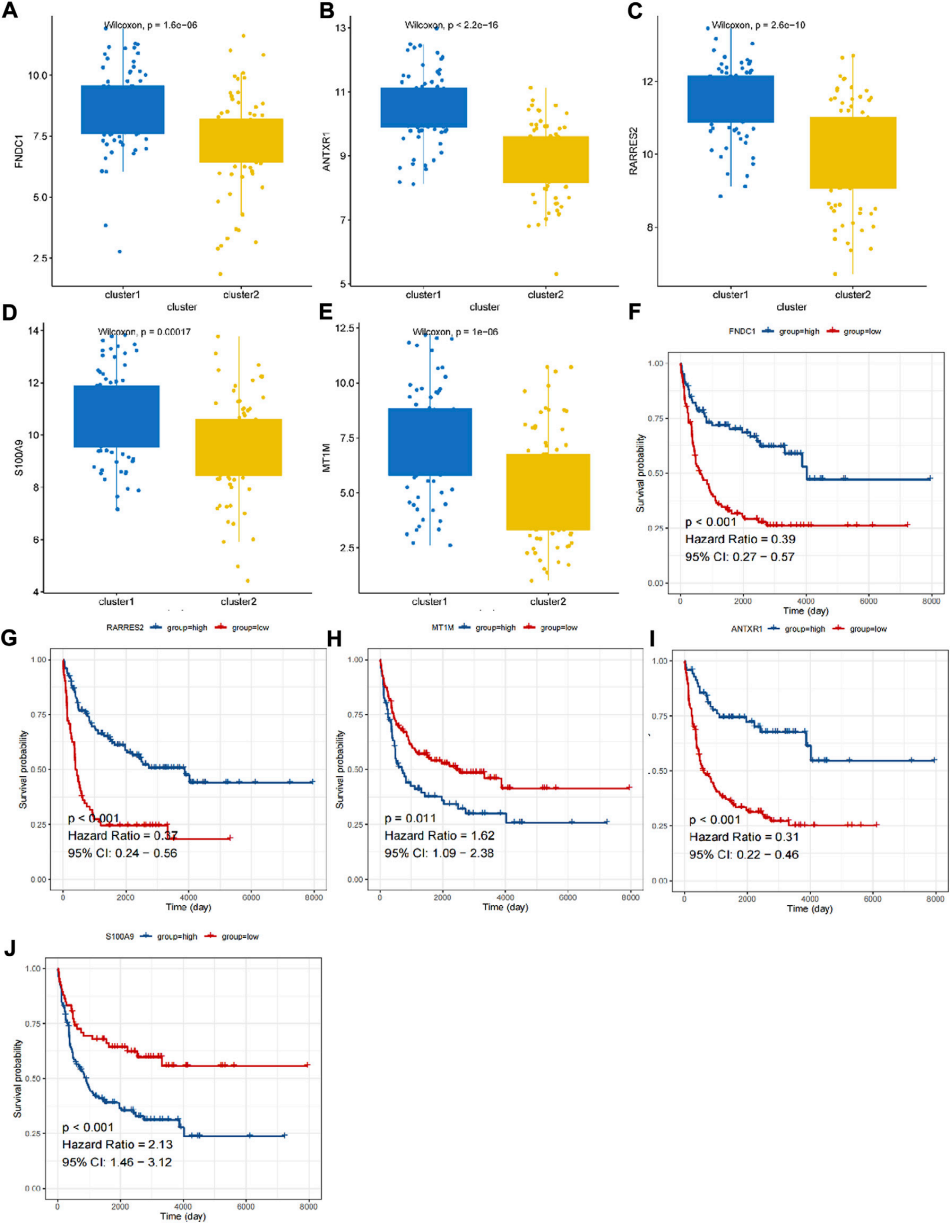
The expression and K-M curves for five prognostic genes. **(A,F)** FNDC1 **(B,G)** RARRES2 **(C,H)** MT1M **(D,I)** ANTXR1 **(E,J)** S100A9.

A total of 531 DEGs were identified between cluster 1 and cluster 2 (Supplementary Table S2; Figure 2A). The expressions are displayed in Figure 2B. Functional analysis showed that they made significant enrichment with 1,367 GO terms (Supplementary Table S3) and 34 KEGG pathways (Supplementary Table S4). The top GO terms and KEGG pathways were relevant to ECM and inflammation, such as extracellular structure organization, ECM–receptor interaction, complement and coagulation cascades, and focal adhesion (Figures 2C,D). After univariate Cox regression analysis, 130 DEGs were found to be related to the survival of DLBCL (Supplementary Table S5). Next, FNDC1, ANTXR1, RARRES2, S100A9, and MT1M were identified as a prognostic signature using the LASSO algorithm (Figures 2E,F). All of them were expressed much higher in cluster 1 than in cluster 2 (Figures 3A–E), and patients with higher expressions of FNDC1, RARRES2, and ANTXR1 and lower expressions of MT1M and S100A9 had better survival (Figures 3F–J).

### A risk model and a nomogram were developed for DLBCL

**FIGURE 4:**
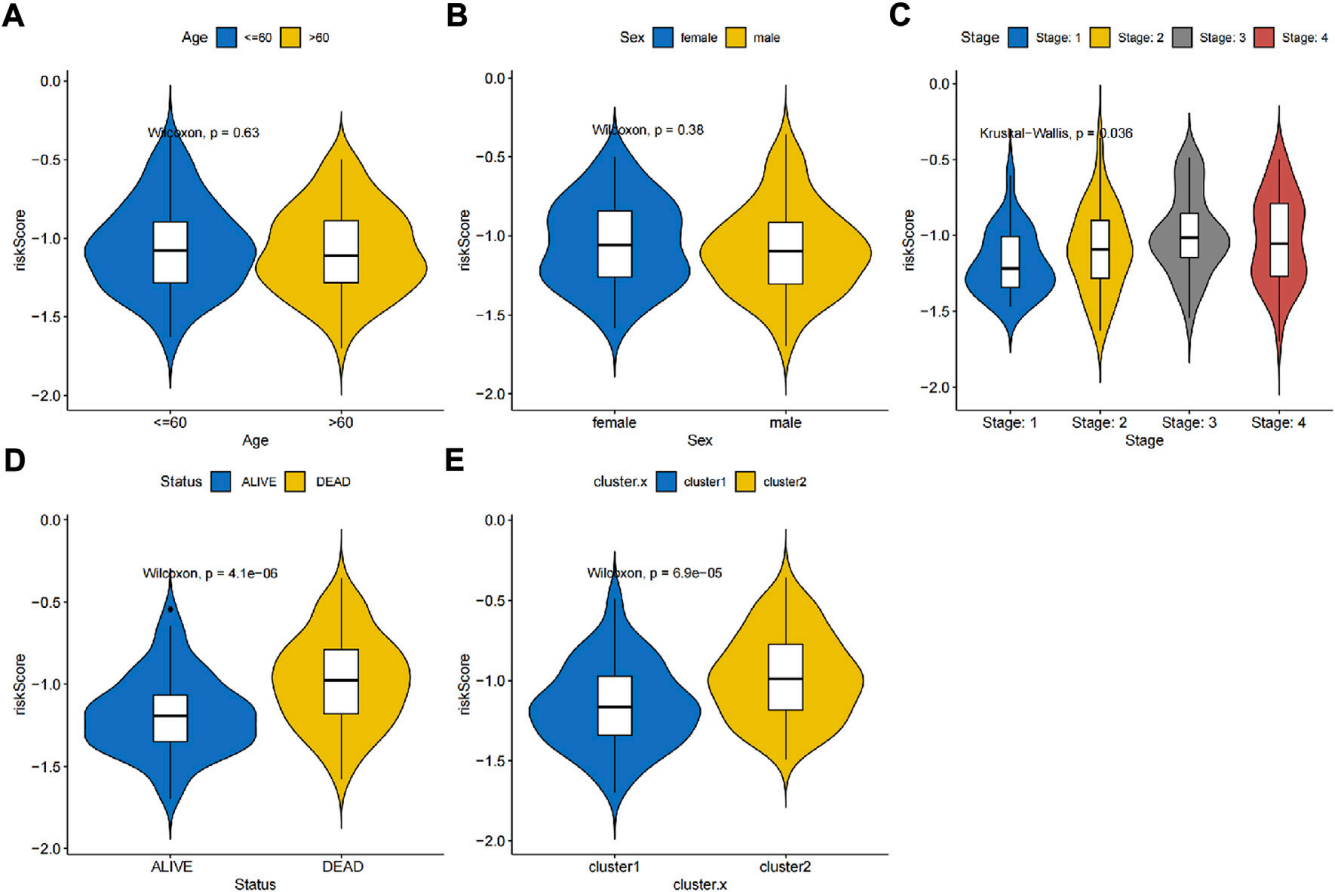
Association between independent prognostic signatures and clinical characteristics. Univariate and multivariate analyses of independent prognostic factors such as **(A)** age, **(B)** sex, **(C)** stage, **(D)** living status and **(E)** clusters developed in this study.

**FIGURE 5:**
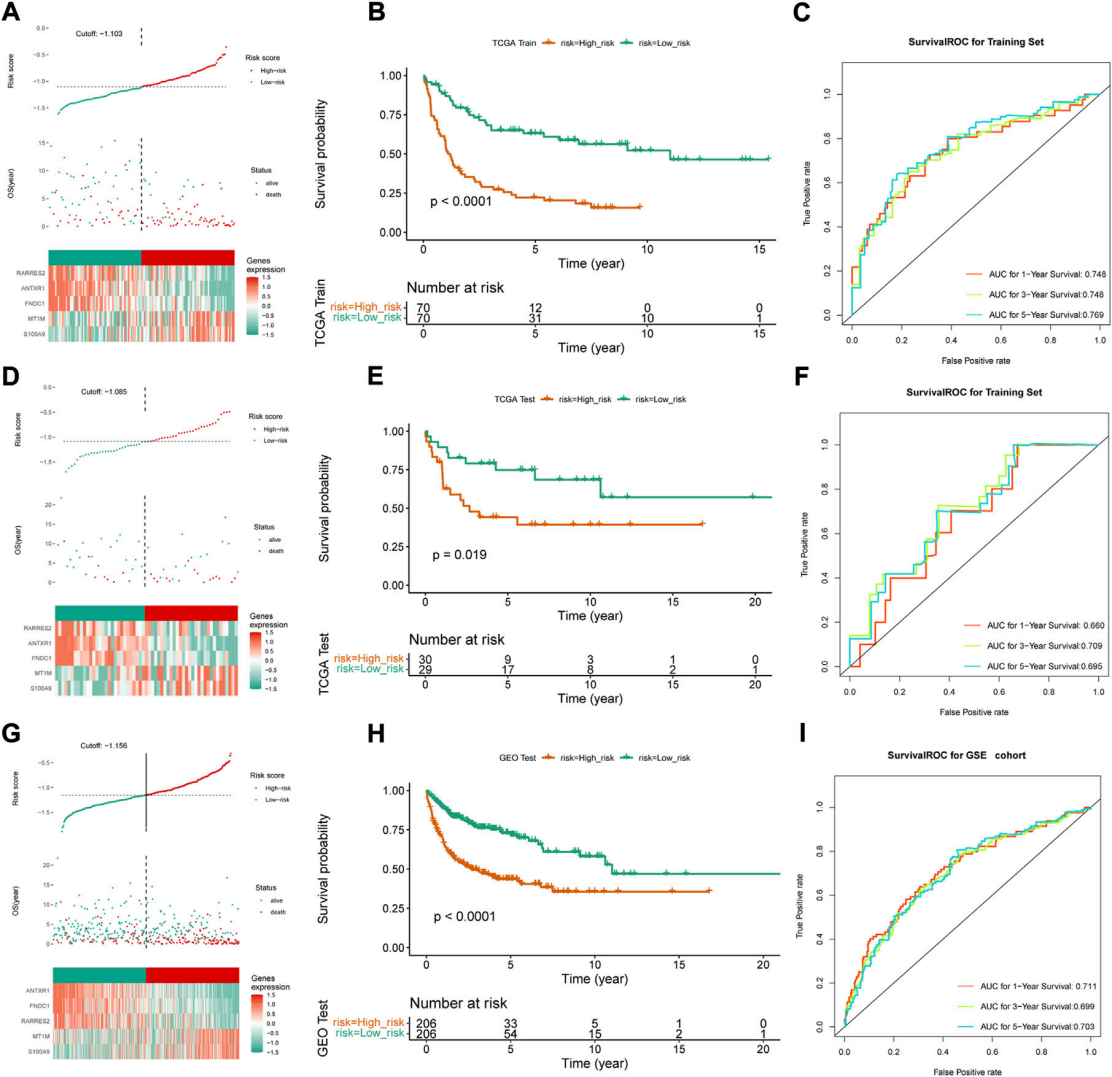
Construction and validation of risk score model. **(A,D,G)** Median risk score in the training and validation set, **(B,E,H)** Survival curve and the median risk score in the training set **(C,F,I)** ROC curves of 1-, 3- and 5-year survival prediction of DLBCL patients in training and validation set.

**FIGURE 6:**
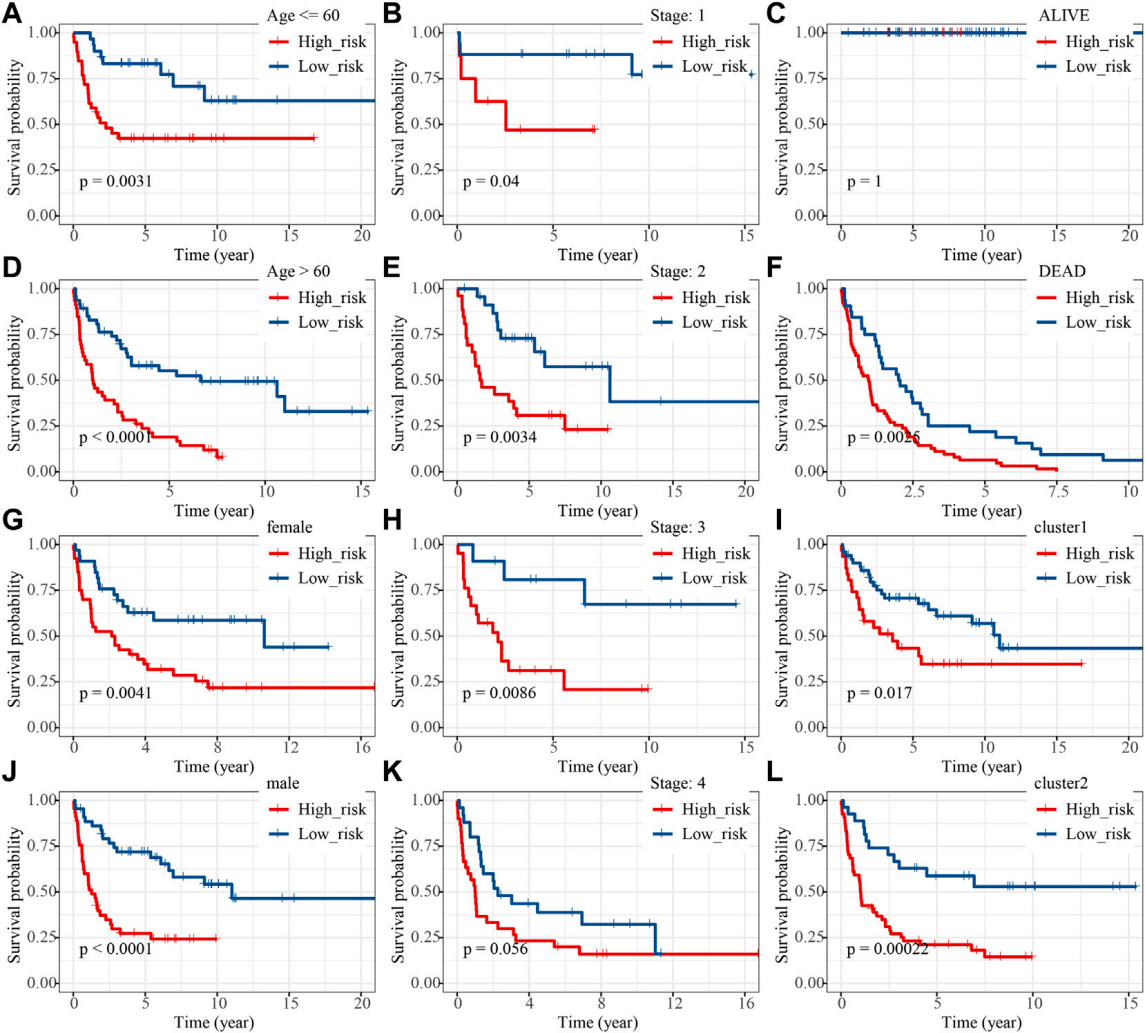
K-M analysis of high- and low-risk groups under different clinical features including **(A,D)** age , **(B,E,H,K)** stage, **(C,F)** living status, **(G,J)** sex and **(I,L)** developed groups.

**FIGURE 7:**
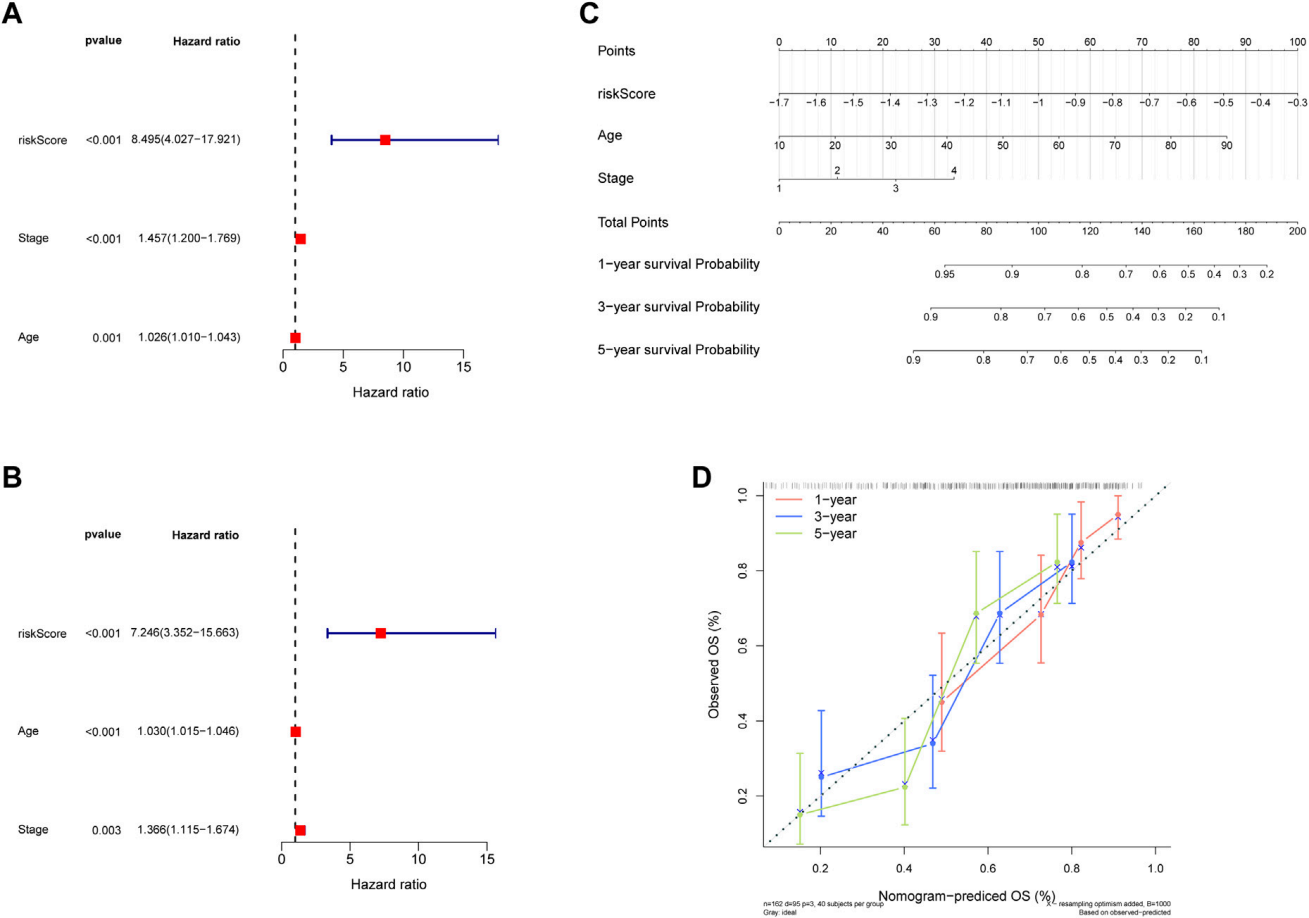
Construction of the nomogram. **(A,C)** Regression analysis and multivariate Cox regression analysis regarding age and stage of 5 hypoxia-related genes signature in training and validaton sets. **(B)** The nomogram to predict the 1-year, 3-year, and 5-year overall survival rate of DLBCL patients. **(D)** The calibration curve for evaluating the accuracy of the nomogram model.

Thus, FNDC1, ANTXR1, RARRES2, S100A9, and MT1M were used to construct the risk model. We found that the risk score were significantly different among groups divided by the tumor stage, living status, and expressions of survival-related HRGs (Figures 4A–E); simultaneously, a heatmap for the expression profile of five biomarkers in different clinical subtypes is presented in Supplementary Figure S1. According to the median of the risk score, patients in the GSE11318 training set were assigned into low- and high-risk groups with markedly different survival rates (Figures 5A,B). ROC curves revealed that the risk model could predict DLBCL’s prognosis with areas under curves (AUCs) greater than 0.7 (Figure 5C). Consistent results were obtained in the GSE11318 testing set (Figures 7D–F) and GSE10846 external validation set (Figures 5G–I). Moreover, a significant survival difference between low- and high-risk groups remained in patients stratified by age (≤60 and >60), gender (male and female), tumor stage (stages 1, 2, and 3), expressions of survival-related HRGs (cluster 1 and cluster 2), and living status (alive or dead) (Figure 6). Next, by univariate and multivariate analyses, we found that the age, tumor stage, and risk score may be independent prognostic factors in DLBCL (Figures 7A,B). Based on them, a nomogram was constructed (Figure 7C), the calibration curves of which showed its predicted survival of 1, 3, and 5 years was similar to the actual survival (Figure 7D).

### Different characteristics were observed between high- and low-risk groups

**FIGURE 8:**
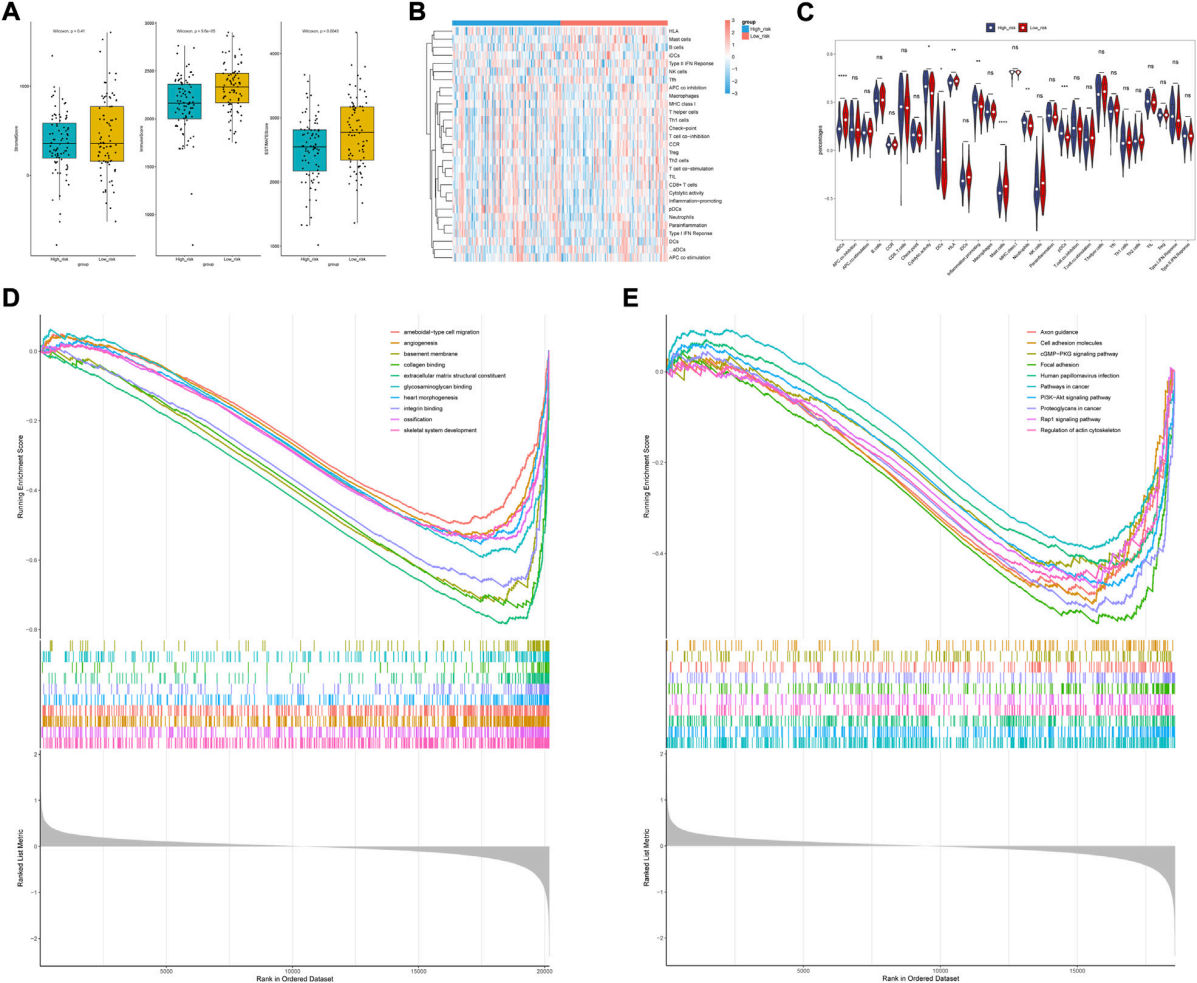
Analysis of immune cell infiltration landscape in DLBCL patients. **(A)** Stroma, immune, and ESTIMATE scores in the high-risk and low-risk groups. **(B)** Correlations among prognostic gene signature and differentially distributed immune cells. **(C)** Comparison of immune infiltration between high- and low -risk group. **(D,E)** Biological functional and pathway enrichment analysis base on GESA algorithm.

**FIGURE 9:**
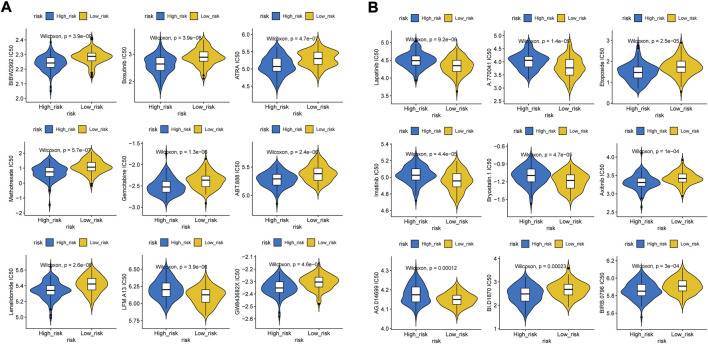
Distribution of IC50 scores. **(A)** Higher IC50 values inclueds Afatinib, Bosutinib, ATRA, Methotrexate, Gemcitabine, Veliparib, Lenalidomide, GW843682X, **(B)** Lower IC50 values includes Etoposide, BI-D1870, Rucapatib, LFM-A13, A-770041, Axitinib, Lapitinib, Imatinib, Bryostatin.1, Doramapimod.

We found that the low-risk group had higher immune and ESTIMATE scores (Figure 8A), and the abundance of aDCs, cytolytic activity, DCs, HLA, inflammation promoting, mast cells, neutrophils, and pDCs was also significantly different between the two groups (Figures 8B,C). The results of the correlation analysis showed that the inflammation-promoting process was closely associated with S100A9 (Supplementary Figure S2). In addition, GSEA also showed that ECM-related GO terms and KEGG pathways were significantly enriched in the low-risk group, such as basement membrane, integrin binding, and focal adhesion (Figures 8D,E). These results suggested that the two groups had different tumor microenvironments. The close relationship between the tumor microenvironment and therapeutic response has been widely reported (Wu and Dai, 2017; Wang et al., 2018; Bejarano et al., 2021). Therefore, we compared the therapeutic response between the two groups. We found that patients had significant differences in sensitivity to 33 drugs between the two groups (Supplementary Tables S6, S7). Herein, we displayed 18 drugs that had significantly different IC50 values between the two groups (Figures 9A,B). In addition, the partial scatter plots of close correlations among five diagnostic genes with those drugs are displayed in Supplementary Figures S3, S4, revealing that the therapeutic response may mainly affect ANTXR1 in DLBCL progress.

## Discussion

Due to its highly heterogeneous characteristics, it is urgently needed to further explore potential novel prognostic biomarkers and the molecular mechanisms of DLBCL. The hypoxia microenvironment and oxidative stress are reported to be closely related to the occurrence, development, treatment, and prognosis of lymphoma. In the hypoxic tumor microenvironment, hypoxia-inducible factor 1 (HIF-1) and its pathway play roles in suppressing the innate and adaptive immune systems to evade immune attack by inducing the expression of immunosuppressive factors and immune checkpoint molecules (Hu et al., 2021; You et al., 2021). The hypoxia-associated risk score model is reported to be associated with poor prognosis through the immunosuppressive microenvironment and immune escape mechanisms (Pei et al., 2021). Meanwhile, Wang et al. (2023) have reported the therapeutic properties of reactive oxygen species for its negative regulation on tumor. Studies on hypoxia and the hypoxia-associated pathway play roles in antitumor regulation and may predict immunotherapy outcomes in an era of machine learning and computational biology (Abou Khouzam et al., 2022). In this work, we aimed to identify novel targets between hypoxia and DLBCL.

We classified the DLBCL patients into two clusters by HRGs. The extracellular matrix (ECM) is known to play roles in supporting the cells and regulating intercellular interactions (Cioroianu et al., 2019). The DEGs between two subtypes were significantly enriched in ECM and inflammation, which support its role in disease development, progression, and response to treatment of DLBCL during hypoxia (Kotlov et al., 2021). Single DLBCL cells play a role in adhesion to adjacent mesenchymal stromal cells and extracellular matrix (Dus-Szachniewicz et al., 2018). Pan et al. reported the extracellular matrix-associated protein SPARC was highly expressed in DLBCL and might be a favorable prognostic biomarker for DLBCL (Pan and Liu, 2021). In addition, fibroblast and extracellular matrix components which represent stromal genetic signatures are associated with good survival in DLBCL (Haro and Orsulic, 2018).

FNDC1, ANTXR1, RARRES2, S100A9, and MT1M were found to be related to prognosis of DLBCL in this study. FNDC1 (fibronectin type III domain containing 1) was found to be related to hypoxia (Zhao et al., 2022) and to be associated with chemoradiation resistance and poor prognosis of gastric cancer, breast cancer, and colorectal cancer by multiple pathways (Ren et al., 2018; Zhong et al., 2018; Liu et al., 2019; Wei et al., 2021; Yunwen et al., 2021; Chen et al., 2022). ANTXR1 is a receptor for anthrax toxin and is highly expressed in tumor endothelial cells. It has been reported to be an oncogene and plays roles in tumor angiogenesis and in the growth, metastasis, and immunosuppression of many kinds of tumors (Huang et al., 2020; Sun et al., 2021). Its antibody–drug conjugate may facilitate selective destruction of tumor blood vessels yielding enhanced anti-cancer efficacy and reduced normal tissue toxicities (Frankel et al., 2011). RARRES2 could initiate chemotaxis *via* the ChemR23 G protein-coupled seven-transmembrane domain ligand which was widely reported to be associated with ischemic hypoxia disease (Zhang et al., 2019; Quan et al., 2021; Jiang et al., 2022). Downregulation or loss of chemerin/RARRES2 in malignancies can modulate the tumor microenvironment and tumor immune responses and act as both a pro- and anti-inflammatory mediator compared to the normal tissue counterparts (Shin et al., 2018). Inhibition of VEGFR1 can result in S100A8/S100A9-mediated calcium influx to induce an M1-like phenotype that impairs ischemic muscle revascularization and perfusion recovery (Ganta et al., 2019). The hypoxia status is positively related to the expression of S100A8/A9 (Grebhardt et al., 2012) which can induce the downregulation of tumor growth and PD-L1 expression through ERK1/2 signaling in NK/T-cell lymphoma. Şeyma Şumnu et al. took a retrospective study by comparing the expression of the S100A8/A9 level between 33 Hodgkin lymphoma (HL) patients and 20 healthy volunteers through ELISA and found it as a biomarker of inflammation in HL. Metallothionein (MT1M) belongs to a family of cysteine-rich cytosolic proteins and plays important roles in metal homeostasis and protection against heavy metal toxicity, DNA damage, and oxidative stress (Si and Lang, 2018; Li et al., 2021b). It acts as a tumor suppressor through upregulation of ROS levels and downregulation of SOD1 (a superoxide dismutase 1) activity and phosphorylation of the SOD1 downstream pathway PI3K/AKT. In this study, we constructed a risk score model based on these five genes, and there were few reports about these genes with DLBCLs. Among them, S100A9 has been reported to be related to other kinds of lymphoma.

We analyzed and compared the tumor microenvironment between the high- and low-risk groups based on the ESTIMATE algorithm. We found that patients with higher immune and ESTIMATE scores had better prognosis but no significant differences in stromal scores. In addition, cytolytic activity, DCs, the inflammation-promoting process, neutrophils, and pDCs were clustered into the high-risk group which may provide novel immunotherapy targets which means the hypoxic TME may play a crucial role in pathogeneses and progression in DLBCL. Consistent with previous results, ECM-related features such as basement membrane, integrin binding, and focal adhesion were significantly enriched in the low-risk group based on GO and KEGG pathway analyses, indicating that these factors may be associated with better prognosis (Hou et al., 2022). We further speculated the possible therapeutic targets using the pRRophetic package and found that higher IC50 values of afatinib, bosutinib, ATRA, methotrexate, gemcitabine, veliparib, lenalidomide, GW843682X, etoposide, BI-D1870, and rucaparib, as well as reduced IC50 values of LFM-A13, A-770041, axitinib, lapitinib, imatinib, bryostatin.1, and doramapimod, were observed in the low immune score group which indicated that patients with a higher immune score presented higher sensitivity to afatinib, bosutinib, ATRA, methotrexate, gemcitabine, veliparib, lenalidomide, GW843682X, etoposide, BI-D1870, and rucaparib, as well as lower sensitivity to LFM-A13, A-770041, axitinib, lapitinib, imatinib, bryostatin.1, and doramapimod, and thus show better therapy sensitivity. As TKIs are constantly being released, drug resistance is still inevitable. Nowadays, immune checkpoint inhibitors have dramatically changed the prognosis of patients. It needs to be verified in subsequent experiments.

Taking all these into consideration, our research revealed five hypoxia-related genes associated with the tumor microenvironment of DLBCL and provided a prognostic prediction model. In addition, we explored the potential immune-related mechanisms of these five genes in regulating DLBCL. Despite a strong association between the new risk model and survival outcomes, our study has several limitations for further verification in gene and protein levels of clinical samples. Our findings enriched the understanding of DLBCL etiology, which will contribute to good clinical practice in immunotherapy and future therapeutic sensitivity research.

## Data Availability

The datasets presented in this study can be found in online repositories. The names of the repository/repositories and accession number(s) can be found in the article/Supplementary Material.
