# Supplementary material for: Hypoxia-related tumor environment correlated with immune infiltration and therapeutic sensitivity in diffuse large B-cell lymphoma
Source: Front Genet. 2022 Oct 14;13:1037716. doi: 10.3389/fgene.2022.1037716 (PMC9614142; doi:10.3389/fgene.2022.1037716)
Supplement: Supplementary file 2 [file DataSheet1.DOCX]

**Supplementary Figures**

**
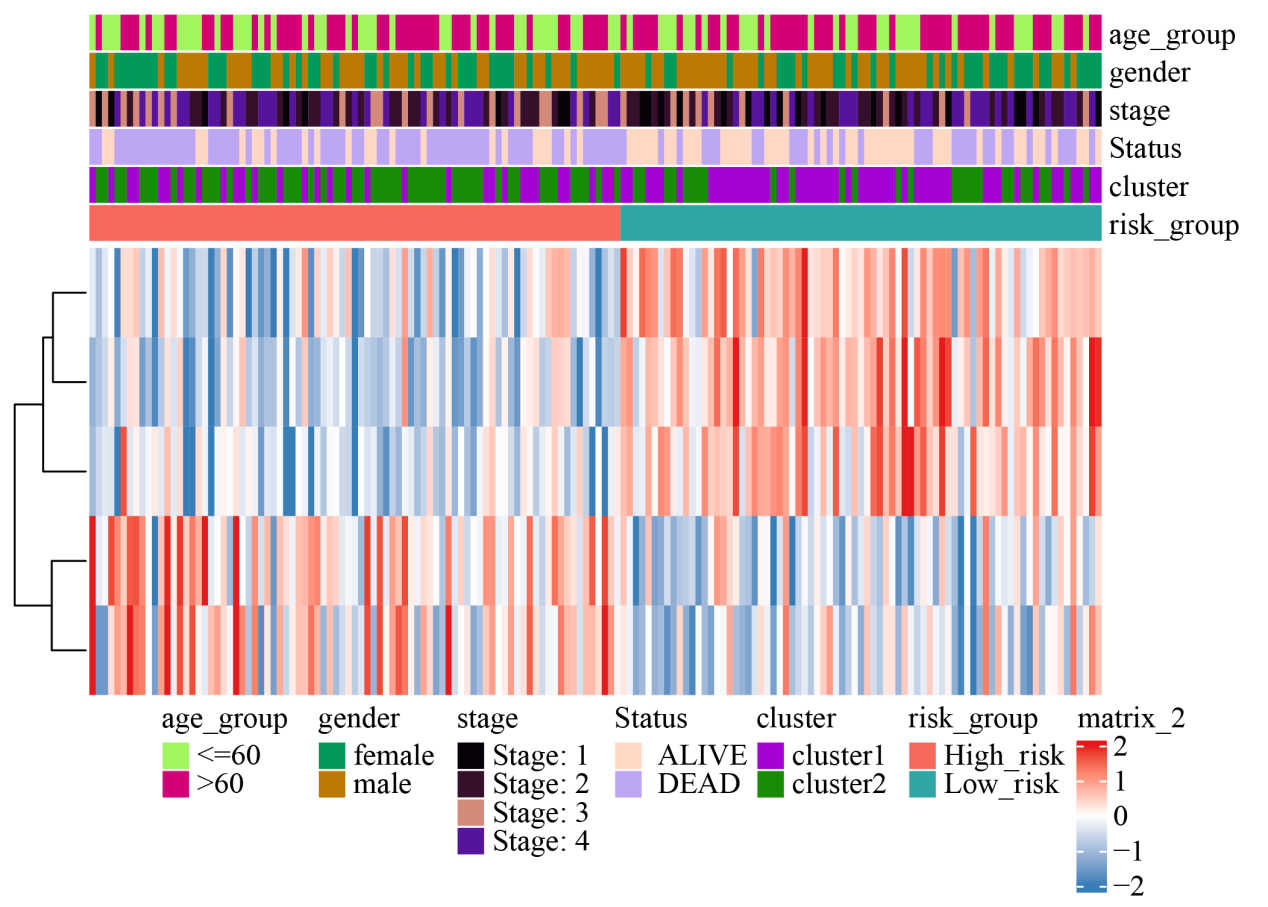
**

**Supplementary Figure 1.** Comprehensive analysis of the differences among different clinical characters.


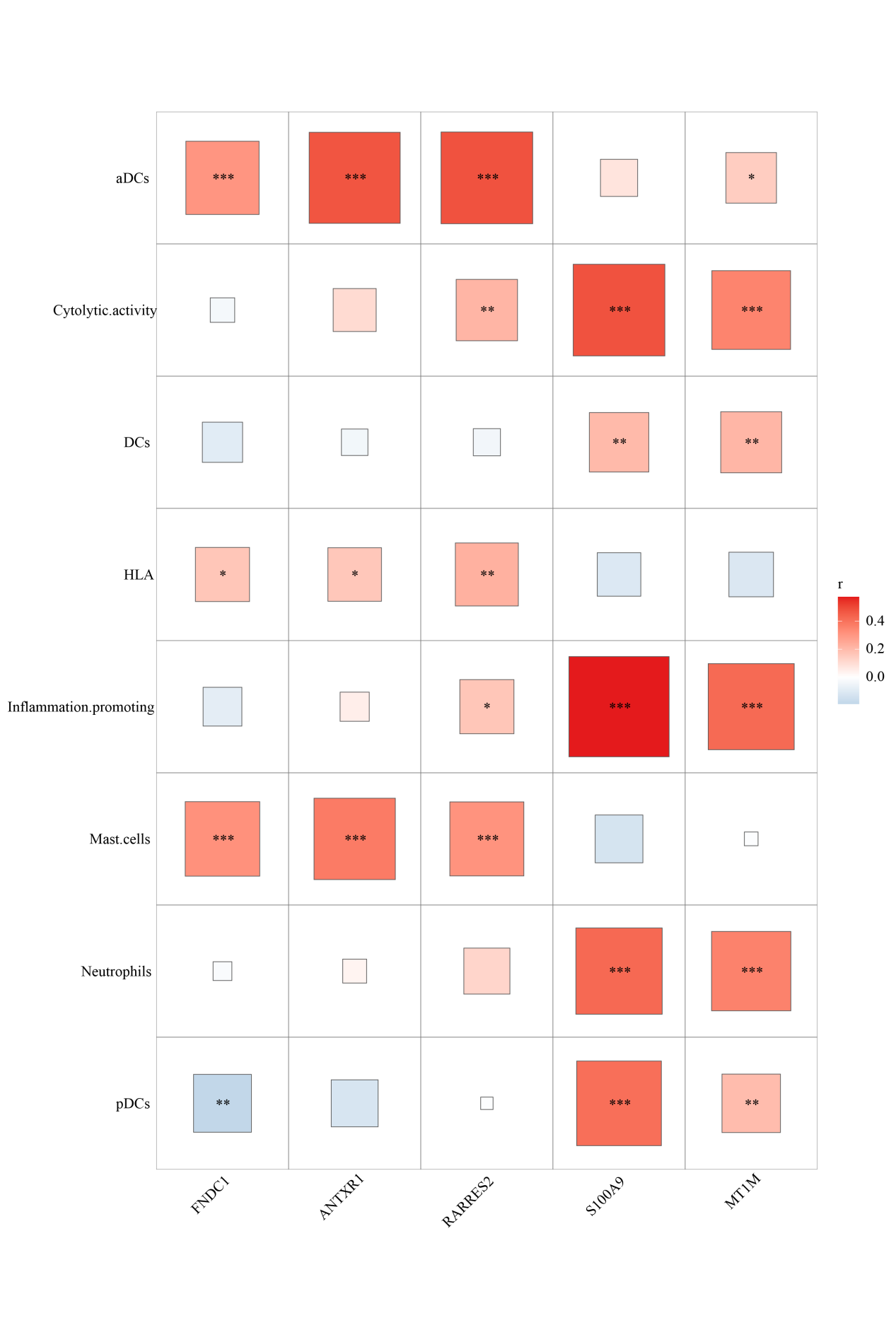


**Supplementary Figure 2.** Correlations among five prognostic genes and differentially distributed immune cells.


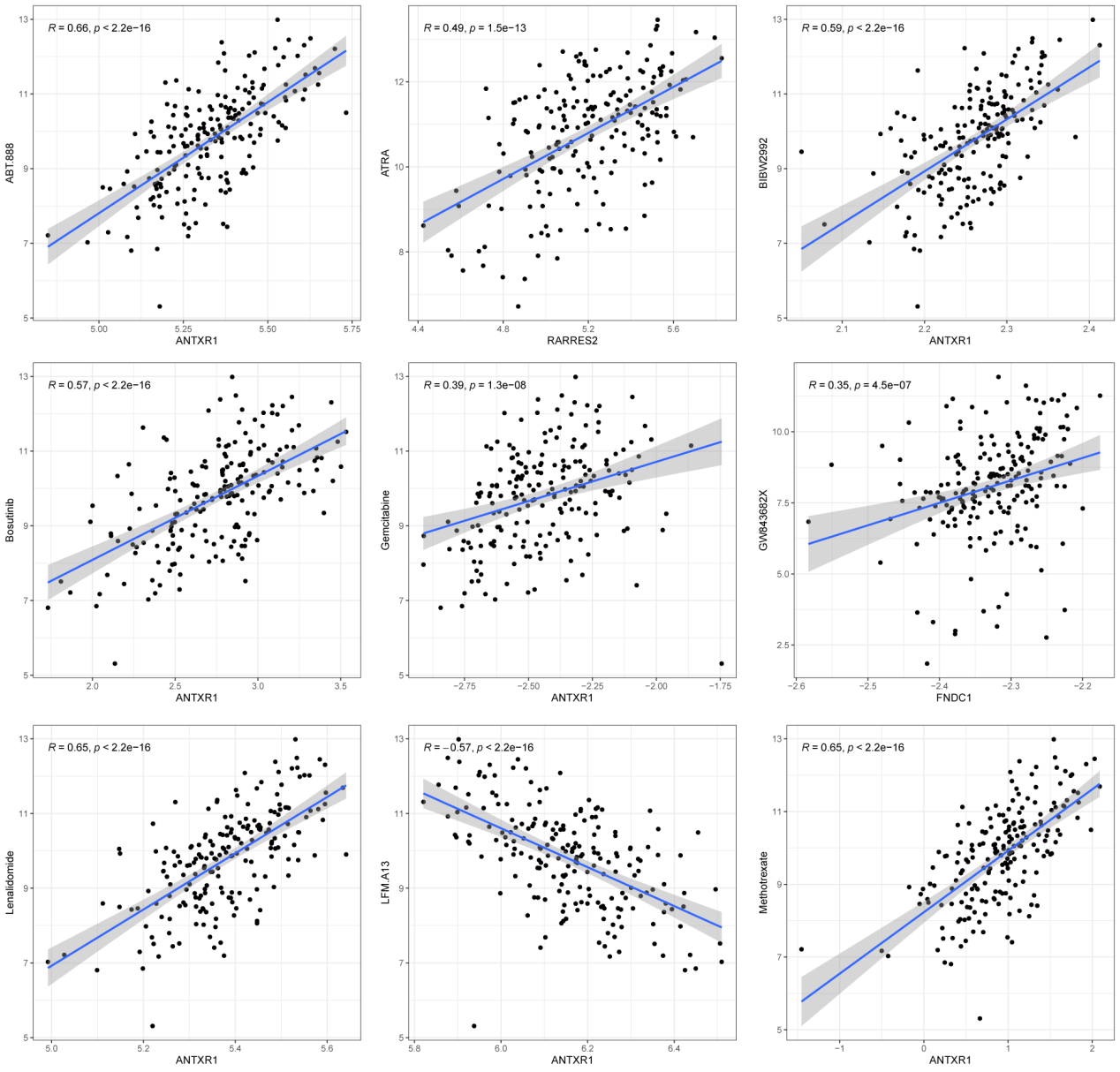


**Supplementary Figure 3.** The correlations between significantly different drugs in high-risk group and five prognostic genes.


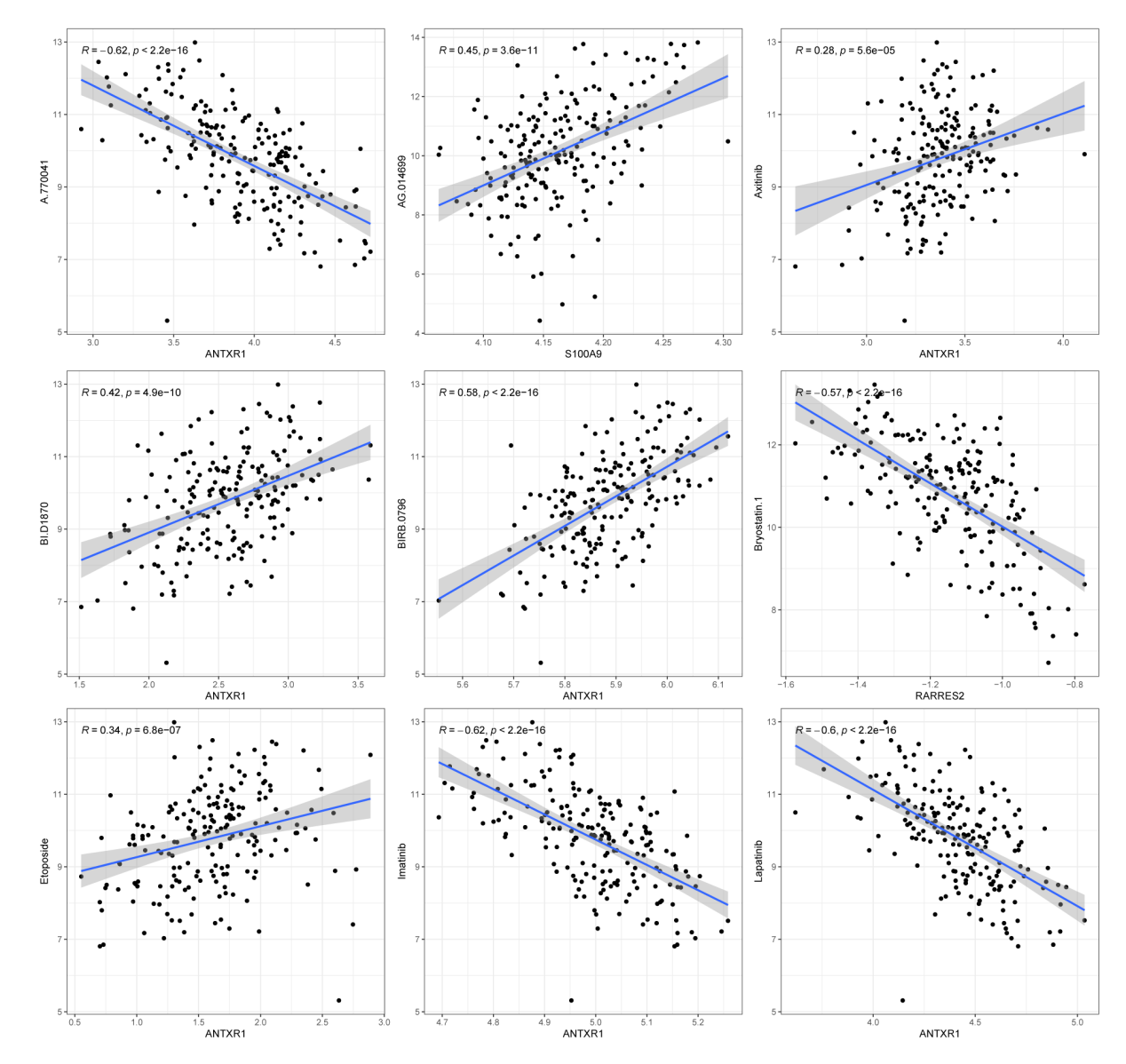


**Supplementary Figure 4.** The correlations between significantly different drugs in low-risk group and five prognostic genes.
